# Effects of ulinastatin and docetaxel on breast cancer invasion and expression of uPA, uPAR and ERK

**DOI:** 10.1186/1756-9966-30-71

**Published:** 2011-07-29

**Authors:** Jie Luo, Xin Sun, Feng Gao, Xiaoliang Zhao, Biao Zhong, Hong Wang, Zhijun Sun

**Affiliations:** 1 Department of Breast, Pancreas, and Thyroid Surgery; Second Affiliated Hospital of Chongqing Medical University, 74 Lingjiang Road, Yuzhong District, Chongqing 400010, China

## Abstract

**Objective:**

To investigate the effects of ulinastatin and docetaxel on invasion of breast cancer cells and expression of uPA, uPAR and ERK, breast cancer MDA-MB-231 and MCF-7 cells.

**Methods:**

The nude mice were treated with PBS, ulinastatin, docetaxel, and ulinastatin plus docetaxel, respectively. Their effects on 1) cell invasion ability was assayed using Transwell; 2) expression of uPA, uPAR and ERK was detected by real time PCR and Western blot; 3) uPA, uPAR and p-ERK protein level in nude mice was quantified by immunohistochemistry.

**Results:**

1) Treatment with ulinastatin, docetaxel, and ulinastatin plus docetaxel, respectively, significantly inhibited MDA-MB-231 and MCF-7 cell invasion; 2) mRNA and protein levels of uPA, uPAR and ERK1/2 were inhibited by ulinastatin, but enhanced by docetaxel.

**Conclusion:**

Ulinastatin can enhance the effects of docetaxel on invasion of breast cancer cells. And that uPA, uPAR and p-ERK expression is obviously inhibited by ulinastatin.

## Introduction

Breast cancer is one of the major malignant tumors threaten women well being. Failure in its treatment mainly arises from cancer proliferation, invasion and metastasis, which ultimately lead to the death of patients. Cell penetrating into extracellular base membrane is the premise of cancer cell metastasis, where a variety of proteases play essential roles.

Plasminogen activators (PAs) are serine proteases, the main function of which is to activate plasminogen into plasmin, a serine protease that hydrolyzes a variety of proteins, including laminin, fibronectin, fibrin, proteoglycan core protein and collagen fibres. There are two types of mammalian PAs: tissue-type (tPA) and urokinase-type (uPA). The former is mainly present in circulatory system, while the latter is present in cells and closely related to tumor cell invasion and metastasis. It has been shown that uPA expression is enhanced in many malignant tumors, such as breast cancer, prostate cancer, colon cancer, stomach cancer and lung cancer, and its mediated-plasminogen activation is dependent on its receptor uPAR in cells. In breast cancer, uPA-uPAR complex is necessary to maintain and amplify plasmin activity[1].

Beside its pivotal roles in pasminogen cascade system, uPA-uPAR complex can also activate many signaling pathways, of which is important Ras-Raf-MEK-ERK pathway. This pathway responds to signals from a variety of growth factors (EGF, NGF, PDGF, etc.), mitogens and environmental stimulations, eventually leading to activation and phosphorylation of extracellular signal-regulated kinase (ERK) through the signal amplification cascade. Phosphorylated ERK translocates to nucleus, where it acts on the AP-1, NF-κB and other nuclear transcription factors, thereby regulating gene expression and promoting tumor cell proliferation, differentiation and survival. Over-activation of ERK has been found in many human malignant tumors including oral cancer, melanoma and breast cancer[2,3].

Urinary trypsin inhibitor ulinastatin as a broad-spectrum protease inhibitor can inhibit trypsin, chymotrypsin, plasmin, human leukocyte elastase and hyaluronidase. It has anti-tumor metastasis and protective effects on patients accepted radiotherapy and chemotherapy and been widely used to treat acute pancreatitis and shock and to improve surgical outcome in clinic. Ulinastatin can bind to tumor cells through its N-terminal Domain I and exert its inhibitory effect on proteolytic activity of plasmin by binding to tumor cells through its C-terminal domain II, the major anti-fibrinolytic group. The impact of ulinastatin on uPA is more complicated. In addition to its inhibitory effects on gene transcription, it also inhibits uPA protein expression by affecting kinase C and MEK/ERK/c-Jun signaling pathways[4,5].

To find a more effective treatment for breast cancer, this study explored the additive effects of docetaxel and ulinastatin on the proliferation of breast cancer MDA-MB-231 cells and tumor growth in nude mice.

## Materials and methods

### 1. Materials

Ulinastatin was purchased from Guangdong Techpool Bio-Pharma Co., Ltd. Docetaxel was bought from Sanofi-Aventis (French). SYBR Green/ROX qPCR Master Mix (2X) were purchased from Fermentas Inc. (Canada). Anti-uPA antibody was from Bioworld (USA). Anti-uPAR and anti-pERK antibodies were from Santa Cruz (USA). 24 well Transwell plates were from Corning (USA). Matrigel was from BD Company (USA).

### 2. Cell culture

Human breast cancer cell line MDA-MB-231 (ER-) and MCF-7 (ER+) were kindly gifted by Shanghai Institute of Biological Sciences, Chinese Academy of Sciences, and maintained in RPMI-1640 medium supplemented with 10% fetal bovine serum, 100 U/mL penicillin, 100 mg/L streptomycin at 37°C in an incubator supplemented with 5% CO_2 _under saturated humidity.

### 3. Animals

100 female BALB/c (nunu) mice at age 4-6 weeks and with body weight of 17-21 g from Animal Research Center of Chongqing Medical University (Production License No.: SCXK (Beijing) 2005-0013, the use permit number: SYX (Chongqing) 2007-0001) were kept in SPF-class environment at 22-25°C and 50-65% humidity. Drinking water, feed and experimental materials were sterilized and all experiments were complied with sterile principle.

### 4. Animal experiments

MDA-MB-231 cells at logarithmic growth phase were washed twice with PBS and prepared as 2.5 × 10^10 ^cells/L suspension in serum-free RPMI-1640 medium. 0.2 mL cell suspension was subcutaneously inoculated in the right armpit of each mouse. 21 days after inoculation, 29 out of 50 mice had tumor volume ≥ 500 mm^3 ^and randomly assigned into 4 groups[6]. MCF-7 cell was innoculated into the other 50 nude mice for building the model[7].

### 5. MDA-MB-231 and MCF-7 cell invasion assay

Breast cancer cell invasion was measured using Transwell chamber. In detail, 2 × 10^5 ^cells were placed in the upper chamber of Transwell with a membrane coated with Matrigel. 24 h later, cells were incubated with 800 U/mL ulinastatin, 3.7 μg/mL docetaxel, 800 U/mL ulinastatin plus 3.7 μg/mL docetaxel, and PBS, respectively, at 37°C in an incubator supplemented with 5% CO_2_. 24 h later, cells in the upper chamber were removed with a cotton swab. The remaining cells on the membrane were stained with 0.1% crystal violet solution and washed with PBS. Crystal violet attached to the cells was dissolved by adding 500 μL of 33% acetic acid into the lower chamber and its absorbance at 570 nm was measured and used to calculate relative amount of cells invaded through the Matrigel to the lower chamber.

### 6. mRNA levels of uPA, uPAR and ERK in MDA-MB-231 and MCF-7 cells measured by real-time RT-PCR

To evaluate the effect of treatments described above on mRNA levels of uPA, uPAR and ERK in breast cancer cells, 24 h after the treatment, total mRNAs were isolated using 1 mL TRIzol reagent according to the protocol provided by the manufacturer. 20 μL mRNA was reverse transcripted into cDNA and the amount of uPA, uPAR and ERK cDNA was examined by quantitative real-time PCR using the following primer pairs: uPA forward primer 5'-GGAGATGAAGTTTGAGGT-GG-3' and reverse primer 5'-GGTCTGTATAGTCCGGG-ATG-3', uPAR forward primer

5'-CACAAAACTGCCTCCTTCCT-3' and reverse primer

5'-AATCCCCGTTGGTCTTACAC-3', ERK forward primer

5'-CCTAAGGAAAAG-CTCAAAGA-3' and reverse primer

5'-AAAGTGGATAA-GCCAAGAC-3', and β-actin forward primer

5'-GCAGAAGGAGATCACAGCCCT-3' and reverse primer

5'-GCTGATCCACATCTGCTGGAA-3'. The corresponding predicted products were 142, 178, 180, and 136 bp, respectively. In detail, template cDNA and primers were mixed with SYBR Green/ROX qPCR Master Mix (2X) in 25 μL reaction system and PCR was carried out in triplicate under the following conditions: 5 min at 95°C, 45 cycles of 15 seconds at 95°C and 30 seconds at 60°C, 1 min at 95°C and 1 minute at 55°C. Ct value of each sample was defined as cycle number when the fluorescence intensity reached the threshold. Relative RNA level was normalized to β-actin and quantified using 2^-ΔΔ^.

### 7. Protein expression of uPA, uPAR and p-ERK1/2 determined by Western blot

24 h after treated as described above, MDA-MB-231 cells were lysed with 25 μL buffer and mixed with 2× sample buffer. Proteins were then subjected to SDS-PAGE and transferred onto PVDF membrane. The membrane was incubated overnight with primary antibodies against uPA, uPAR and p-ERK1/2, respectively, at 4°C and subsequently with secondary antibodies for 1 hour. After wash with PBST, signals were visualized by incubation with ECL luminescence substrate and detected with Universal Hood2 Chem GelDocxR Gel Imaging System (Bio-Rad, USA).

### 8. Expression of uPA, uPAR and p-ERK1/2 in mouse xenografts by immunohistochemistry SP method

uPA, uPAR and p-ERK1/2 in slides of collected mouse xenografts were labeled with antibodies against uPA, uPAR and p-ERK1/2, respectively, followed by incubation with corresponding secondary antibodies. The labeled proteins were visualized with DAB reagent and examined under microscope. Cells with brown or brownish yellow granules were considered as positive and analyzed using Image Pro-plus 6.0 image analysis software to calculate integrated optical density (IOD).

### 9. Statistical analysis

All data were expressed as mean±s and analyzed using statistical analysis software SPSS 18.0. Differences between groups were tested using analysis of variance. A p value less than 0.05 was considered as statistical significance.

## Results

### 1. Effects of ulinastatin and docetaxel on MDA-MB-231 and MCF-7 cells invasion

**Figure 1:**
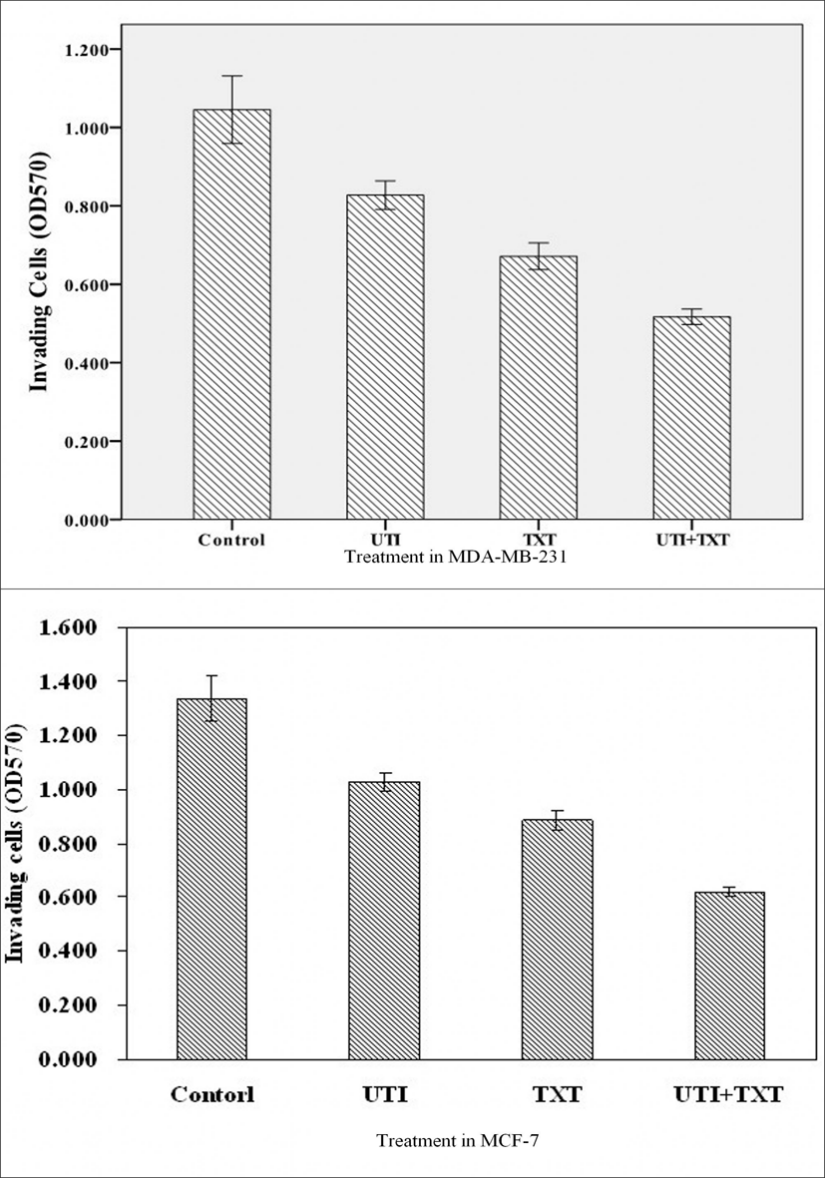
**Inhibition of ulinastatin and docetaxel on MDA-MB-231 and MCF-7 cell invasion**. Shown are the absorptions at 570 nm of cells treated with ulinastatin, docetaxe and ulinastatin plus docetaxe for 24 hours, respectively, in the lower chambers of transwells. Treatment of cells with ulinastatin, docetaxe and ulinastatin plus docetaxe significantly inhibited MDA-MB-231(1a) and MCF-7 (1b) cell invasion.

Absorbance value at 570 nm reflects the number of cells penetrated the Matrigel and membrane of the Transwell. As shown in Figure 1, the invasion rates of cells treated with ulinastatin, docetaxel and ulinastatin plus docetaxel were 20.861%, 35.789% and 52.823%, respectively, all significantly decreased compared with that of the control (p < 0.01).

### 2. Effects of ulinastatin and docetaxel on uPA, uPAR and ERK mRNA level

**Figure 2:**
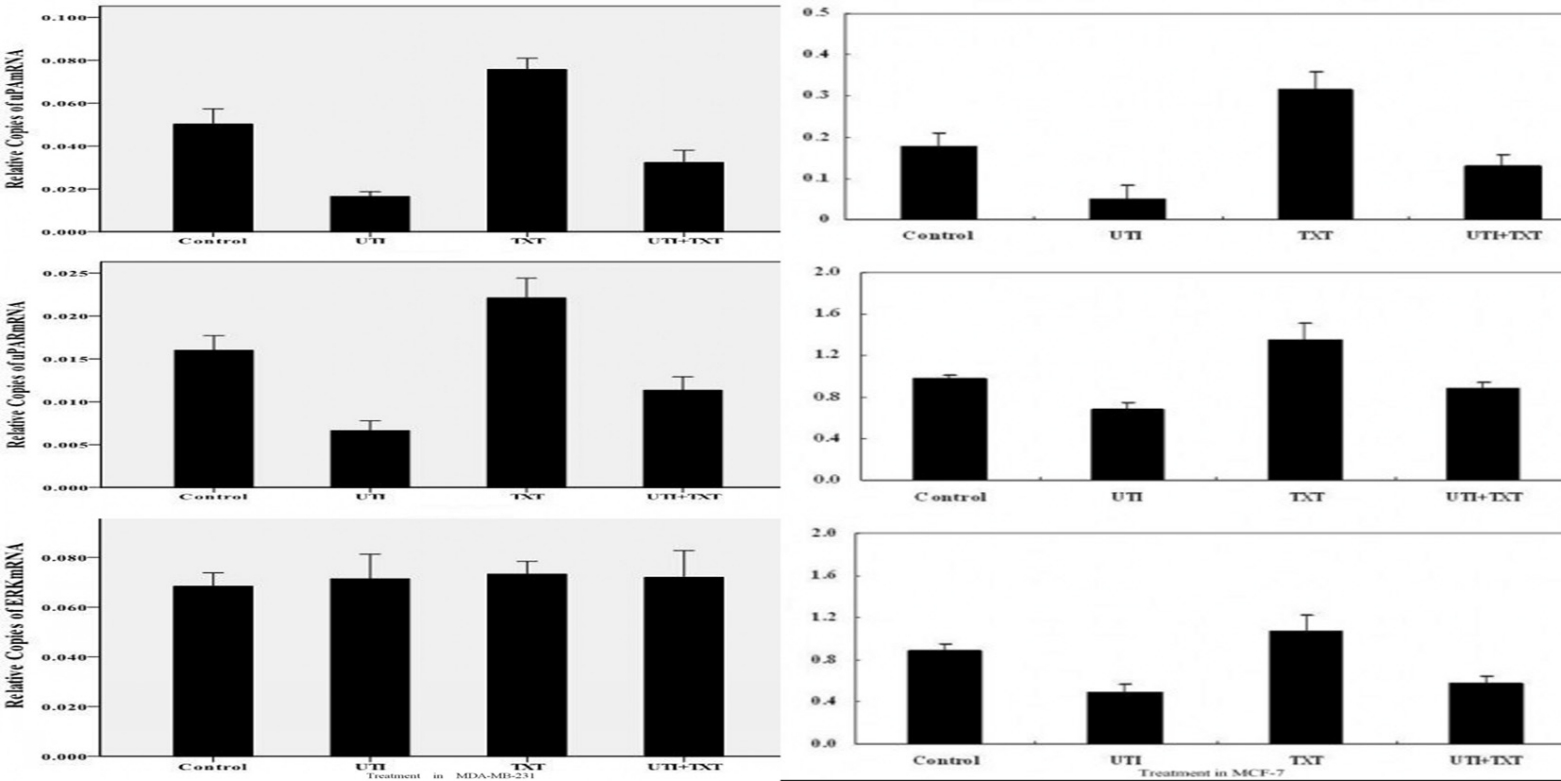
**Effects of ulinastatin and docetaxe on mRNA level of uPA, uPAR and ERK in MDA-MB-231 cells and MCF-7 cells.** （1）Shown are the RT-PCR results of relative mRNA levels of uPA (a) uPAR (b) and ERK (c) to β-actin in MDA-MB-231 cells treated with ulinastatin, docetaxe and ulinastatin plus docetaxe for 24 hours, respectively. (2) Shown are the RT-PCR results of relative mRNA levels of uPA (a) uPAR (b) and ERK (c) to β-actin in MCF-7(a,b,c) cells treated with ulinastatin, docetaxe and ulinastatin plus docetaxe for 24 hours, respectively.

As shown in Figure 2(1), uPA and uPAR mRNA levels in MDA-MB-231cells treated with ulinastatin as well as ulinastatin plus docetaxel were significantly decreased compared with those in control treated cells (p < 0.05). By contrast, uPA and uPAR mRNA levels were significantly enhanced in cells treated with docetaxel (p < 0.05). In addition, all treatments had no effects on ERK mRNA level (p = 0.9). However, ERK mRNA has statistical difference in MCF-7 (p < 0.05). Figure 2(2).

### 3. Effects of ulinastatin and docetaxel on uPA, uPAR and phosphorylated ERK1/2 (p-ERK1/2) proteins

**Figure 3:**
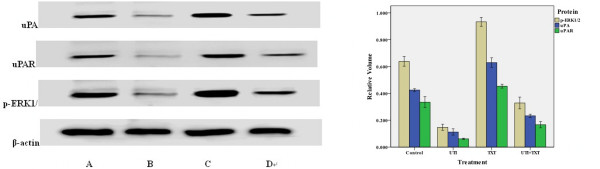
**Effects of docetaxe and ulinastatin on expression of uPA, uPAR and p-ERK1/2 in MDA-MB-231 cells**. (1) Shown are the representative results of western blot of uPA, uPAR and p-ERK1/2 in MDA-MB-231 cells treated with control, ulinastatin, docetaxel, and ulinastatin plus docetaxel, respectively. (2) Shown are the quantitative results of western blot experiments.

Levels of uPA, uPAR and p-ERK1/2 in MDA-MB-231 cells treated with ulinastatin and docetaxel are shown in Figure 3(1). Treatment of cells with ulinastatin alone or along with docetaxel significantly decreased uPA, uPAR and p-ERK1/2 level in MDA-MB-231 cells. By contrast, treatment of cells with docetaxel significantly augmented uPA, uPAR and p-ERK1/2 levels Figure 3(2) (p < 0.05).

### 4. uPA, uPAR and p-ERK1/2 level in exograft of nude mice

**Figure 4:**
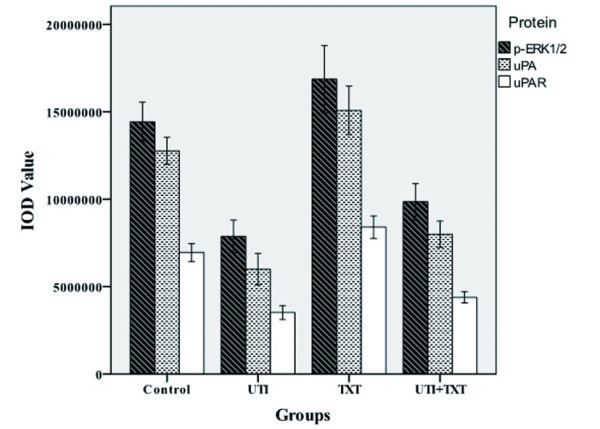
**Effects of docetaxe and ulinastatin on expression of uPA, uPAR and p-ERK1/2 in mouse exografts**. Shown are the quantitative results of uPA, uPAR and p-ERK1/2 expression in exografts of mice treated with control, ulinastatin, docetaxel, and ulinastatin plus docetaxel, respectively, in immunohistochemical experiments.

**Figure 5:**
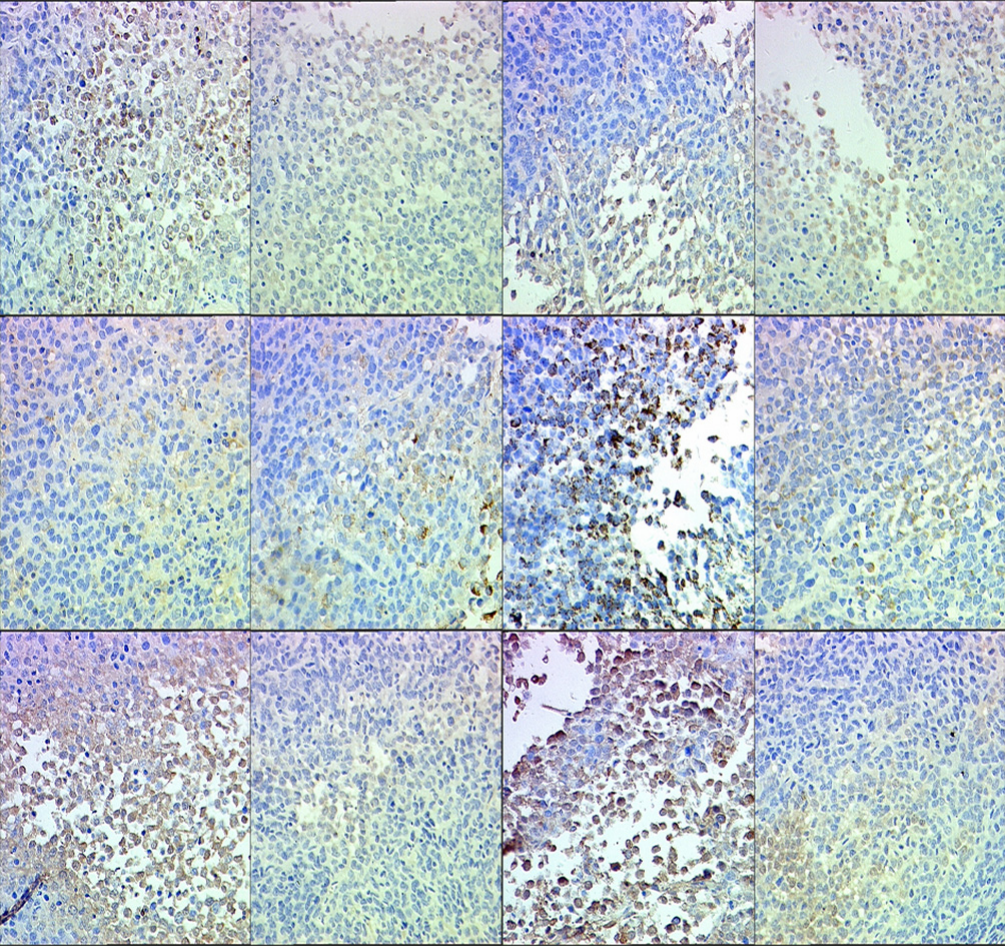
**Positive immunohistochemical expression of uPA, uPAR, p-ERK1/2 in MDA-MB-231 exnografts of mice in control(a), ulinastatin(b), docetaxel(c),ulinastatin plus docetaxel(d) groups (SP,×400）**(1). Positive immunohistochemical expression of uPA in MDA-MB-231 exnografts of mice in control (a), ulinastatin (b), docetaxel (c), and ulinastatin plus docetaxel (d) groups (SP, ×400).(2). Positive immunohistochemical expression of uPAR in MDA-MB-231 exnografts of mice in control (a), ulinastatin (b), docetaxel (c), and ulinastatin plus docetaxel (d) groups (SP, ×400).(3). Positive immunohistochemical expression of p-ERK1/2 in MDA-MB-231 exnografts of mice in control (a), ulinastatin (b), docetaxel (c), and ulinastatin plus docetaxel (d) groups (SP, ×400).

Specimens of MDA-MB-231 mouse exografts were immunostained for uPA, uPAR and p-ERK. The IOD values of the targeted proteins in each group were statistically analyzed. The levels of uPA, uPAR and p-ERK1/2 in ulinastatin group were lower than those of ulinastatin plus docetaxel group; both groups had significant lower levels of uPA, uPAR and p-ERK1/2 than the control group. Figure 4,6. By contrast, the levels of uPA, uPAR and p-ERK in docetaxel group were significantly higher than those of the control group (p < 0.05). The immunohistochemistry result of MCF-7 is same as the result in MDA-MB-231. Figure 5,7.

## Discussion

Proliferation and invasion are important biological features of breast cancer. Because the development of breast cancer involves many extremely complicate regulatory factors, its treatment is often difficult. Therefore, the objective of the study is to explore various cytokines' mechanisms and relationship in regulating tumor cell proliferation and invasion, and eventually find the corresponding optimal therapeutic measures.

Urokinase-type plasminogen activator (uPA) is the hub of the plasminogen activator system, also known as uPA system. As a multifunctional serine protease, in addition to its direct contribution to the degradation of extracellular matrix, uPA also mediates activation of matrix metalloproteinase[7], thereby promoting cancer cell invasion and migration. Recent studies have revealed that uPA is involved in angiongenesis and lymphangiogenesis[8] and related to cell proliferation-related signal transduction pathway. Binding of uPA to its receptor uPAR is known to regulate uPAR expression. Therefore, uPA and uPAR usually are similarly over-expressed in breast cancer cells[9].

Ulinastatin binds to cells through its domain I, and exerts its anti-fibrinolytic activity through its domain II. Our results of real time PCR showed that ulinastatin treatment decreased uPA and uPAR mRNA level, suggesting that ulinastatin can inhibit uPA at genetic level and subsequently reducing the expression of uPAR.

ERK belongs to a class of serine/threonine protein kinases found in late 80s of the last century and is a member of Ras-Raf-MEK-ERK signal transduction pathway. Phosphorylated ERK (p-ERK) can promote cell survival, growth and mitosis by regulating nuclear transcription factor NF-κB activity. The promoter of uPA gene has NF-κB binding sites, therefore, p-ERK can increases expression of uPA through activation of NF-κB[10]. In addition, a large number of studies in recent years have confirmed[2,3,11-13] that binding of uPA to uPAR can activate Ras-ERK pathway.

For example, in human breast cancer MCF-7 cells, when the LDL receptor family members are depolymerized, binding of endogenous uPA to uPAR can activate ERK[14,15]. The result shows in MCF-7 cells either, its ERK decressed obviously. Furthermore, uPAR can also regulate basal p-ERK level by binding to integrin α5β1[3,16]. Therefore, uPA-uPAR and ERK can activate each other through different pathways and form a positive feedback loop, thereby maintaining high proliferating and invasive ability of cancer cells.

**Figure 6:**
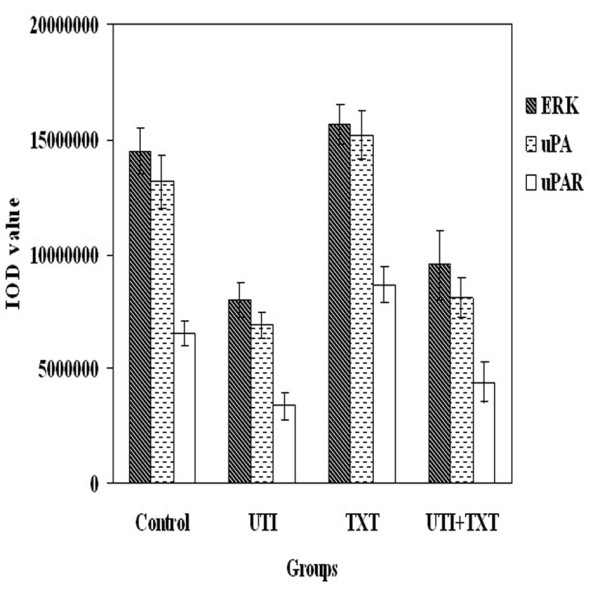
**Effects of docetaxe and ulinastatin on expression of uPA, uPAR and p-ERK1/2 in mouse exografts**. Shown are the quantitative results of uPA, uPAR and p-ERK1/2 expression in exografts of mice treated with control, ulinastatin, docetaxel, and ulinastatin plus docetaxel, respectively, in immunohistochemical experiments.

**Figure 7:**
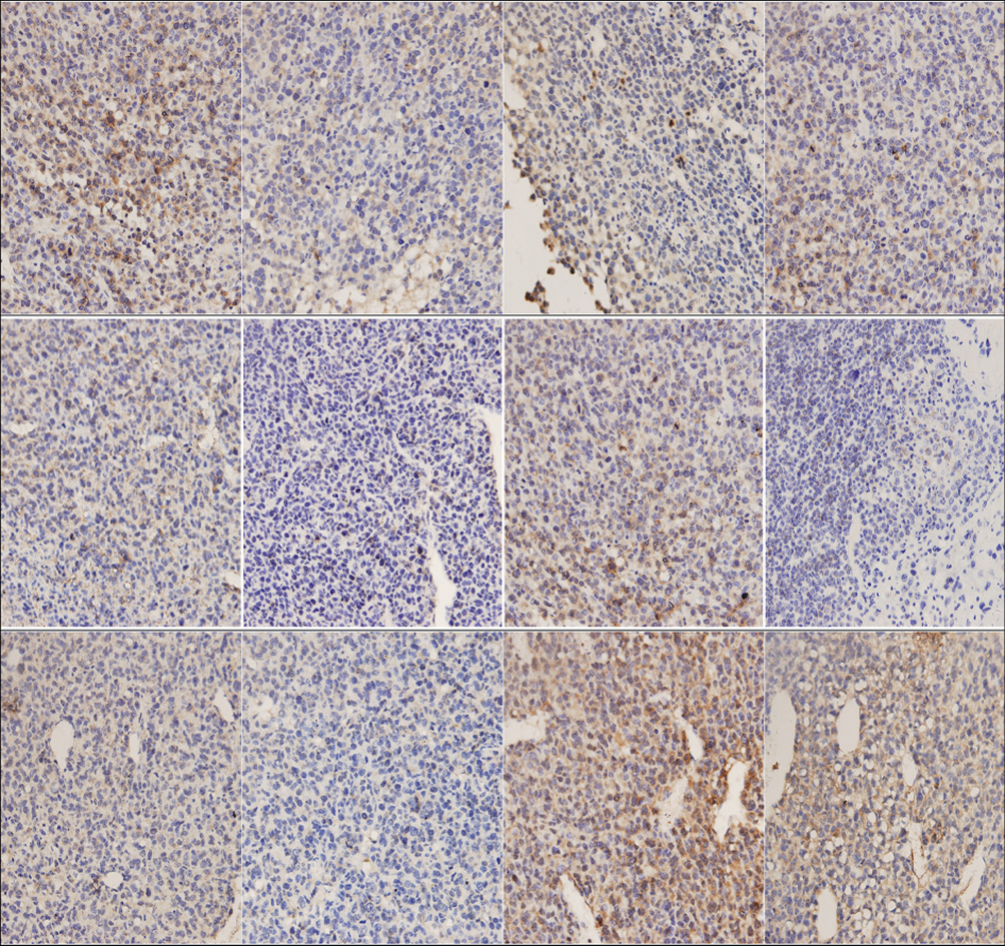
**Positive immunohistochemical expression of uPA, uPAR, p-ERK1/2 in in MCF-7 exnografts of mice in control(a), ulinastatin(b), docetaxel(c),ulinastatin plus docetaxel(d) groups (SP,×400)** (1).Positive immunohistochemical expression of uPA in MCF-7 exnografts of mice in control (a), ulinastatin (b), docetaxel (c), and ulinastatin plus docetaxel (d) groups (SP, ×400). (2) Positive immunohistochemical expression of uPAR in MCF-7 exnografts of mice in control (a), ulinastatin (b), docetaxel (c), and ulinastatin plus docetaxel (d) groups (SP, ×400). (3). Positive immunohistochemical expression of p-ERK1/2 in MCF-7 exnografts of mice in control (a), ulinastatin (b), docetaxel (c), and ulinastatin plus docetaxel (d) groups (SP, ×400).

The basal expression of uPA, uPAR and p-ERK in breast cancer MDA-MB-231 cells are very high[17,18]. Ulinastatin treatment could significantly decrease uPA and uPAR protein expression and mRNA level compared with control group (p < 0.05), possibly due to its inhibitory effect on the translocation of protein kinase C from the cytoplasm to the membrane and consequent down-regulation of MEK/ERK/c-Jun pathway, thereby causing the decline in uPA expression[5]. its mediated-downregulation of uPA inhibited ERK phosphorylation Figure 4,5,6,7.

Docetaxel can cause cancer cell mitotic arrest at G2/M phase by inhibiting tubulin depolymerization and promoting non-functional microtube formation. Further studies in recent years have revealed a role of docetaxel in other mechanisms besides cell toxicity. Our experiments also showed that docetaxel treatment increased p-ERK1/2 level (p < 0.05), but decreased uPA and uPAR mRNA and protein levels (p < 0.05), in consistence with the reports of Yacoub and Mhaidat[19,20]. The specific mechanism on how docetaxel functions has not yet been clarified, but probably is related to its role in initiation of cell apoptosis and consequent activation of ERK pathway and p-ERK-dependent upregulation of uPA expression. In addition, reports have shown that pretreatment of cells with other ERK activity specific inhibitor can markedly promote the effect of docetaxel on cell apoptosis[20,21]. Our study also found that treatment of cells with ulinastatin along with docetaxel significantly inhibited uPA, uPAR and ERK1/2, leading to the maximum cell apoptosis rate among the three treatment groups (83.254% at 72 hours)[6]. Therefore, the upregulation of these three proteins in response to docetaxel treatment should be considered as one of the drug-resistance mechanisms of MDA-MB-231 cells, and application of inhibitors (such as ulinastatin) can weaken this resistance.

This study revealed that uPA, uPAR and p-ERK expression is obviously inhibited by ulinastatin. Because many factors and mechanisms are involved in cancer cell proliferation, although treatment with ulinastatin alone can inhibit MDA-MB-231 cell proliferation and exograft growth[6], its effect is not as strong as that combined with docetaxel. On the other hand, although docetaxel enhanced the expression of uPA, uPAR and ERK1/2, its cell toxicity still plays a dominant role, so when treated with docetaxel alone, the proliferation and tumor growth of breast cancer cell was inhibited. Combined treatment of ulinastatin plus docetaxel is more effective in anti-tumor invasion. Therefore, the role of ulinastatin in the antitumor aspect deserves further study.

## Competing interests

The authors declare that they have no competing interests.

## Authors' contributions

JL did the cell invasion essay and immunohistochemistry, XS did the Cell-culturing, submitted paper and revised the paper, FG did the medical statistics, XZ cultured the cell and did PCR, BZ tested the cells in PCR, HW detected the cells in western blot, ZS designed this experiment and wrote this paper. All authors read and approved this final draft.
